# Palliative care in a rural subdistrict in South Africa: A 4-year critical review

**DOI:** 10.4102/phcfm.v16i1.4047

**Published:** 2024-01-21

**Authors:** Agnes Hamilton-Baillie, Louis S. Jenkins, Margie Munnings, Ernestine Bruinders, Annemarie Bekker

**Affiliations:** 1 Faculty of Improving Global Health, Thames Valley and Wessex Leadership Academy, Winchester, United Kingdom; 2 Department of Family and Emergency Medicine, Faculty of Medicine and Health Sciences, Stellenbosch University, Stellenbosch, South Africa; 3 Department of Primary Health Care Directorate, Family, Community and Emergency Care, Faculty of Health Sciences, University of Cape Town, Cape Town, South Africa; 4 Department of Family and Emergency Medicine, George Hospital, Western Cape Department of Health, George, South Africa; 5 Department of Physiotherapy, George Hospital, Western Cape Department of Health, George, South Africa

**Keywords:** palliative care, rural model, integrated, review, South Africa

## Abstract

**Background:**

Palliative care (PC) is a priority in South Africa, focussing on integrating PC into primary health care. Few examples exist showing how this is done. In 2018, a rural PC project was implemented, which subsequently evolved into an integrated service between the hospital and the community.

**Aim:**

The aim was to review the PC project over 4 years.

**Setting:**

The setting was the George subdistrict of the Garden Route district in South Africa. Community-based services were offered to all patients with PC needs by three non-governmental organisations who deliver home community-based care via community health workers. They were supplemented by primary health care clinics, an intermediate care facility and two hospitals.

**Methods:**

This was a retrospective descriptive study. Inpatient ward round data and patient referrals between 2018 and 2022 were analysed using descriptive statistics. Variables included patient demographics, diagnosis, home visits and place of death.

**Results:**

A total of 819 patients were referred. Inpatients were reviewed on weekly ward rounds by a multidisciplinary team. The most common diagnosis was cancer (57%). Home visits enabled patient follow-ups, of which 152 were recorded.

**Conclusion:**

The programme has become sustainable and integrated in the public healthcare system. Contributing factors included dedicated staff, using simple tools and continuous training. The findings may be useful to PC programmes in similar contexts elsewhere.

**Contribution:**

This work adds new knowledge to the field of PC in an underresourced rural healthcare environment in sub-Saharan Africa, by describing how system-wide integration of a new service was navigated to become sustainable.

## Introduction

The World Health Organization (WHO) emphasises the global need for good palliative care (PC), supporting patients and families who are facing life-limiting illnesses.^1^ The WHO estimates that 40 million people annually need PC, 78% of them living in low- and middle-income countries. Globally, PC is only available to about 14% of individuals who need it.^1^ Unless addressed, this unmet need will continue to grow due to ageing populations with increasing burdens of disease.

In South Africa, the increasing prevalence of non-communicable diseases such as hypertension, diabetes, malignancies and infectious diseases like human immunodeficiency virus (HIV) infection and tuberculosis drives the need for PC services for those developing end-stage and life-limiting complications.^2^ Traditionally, responsibility for PC has fallen to non-governmental organisations (NGOs), charities and external donors, outside of the public health systems; however, this has led to inequitable, fragmented and limited access to services.^3^ More recently, there has been a shift away from this traditional siloed approach, with a recognition of the importance of integrating sustainable PC into the continuum of health service delivery to increase access and to strengthen health systems.^3^ Nationally, there has been a drive to strengthen PC services at the primary health care (PHC) level, in communities and in the home. The South African National Policy Framework and Strategy on Palliative care (NPFSPC) was published in 2017, in line with WHO resolution 69.17, which calls for member states to develop policy to strengthen PC systems.^4^

Despite a similar increase in PC advocacy and policy globally, many African countries are still in need of PC services and training, with barriers remaining in medication availability, policies, service provision and teaching.^5^ Most PC services are concentrated in a few African countries, including Kenya, Uganda and South Africa.^5^

South Africa is recognised as being the African country with the widest provision of PC.^3^ However, in 2017, the country had only approximately 160 PC services serving only 40 000 people.^3^ Different methods of PC provision have been described in the South African setting, for example, hospital-based PC services,^6^ hospice-hospital partnerships^7^ and hospice-led, community-based home care.^8^

The Western Cape Department of Health has prioritised PC, with the George subdistrict becoming an implementation site for a rural PC model in 2018.^9^ This pilot project has evolved into a fully integrated service extending from the community home-based services and NGOs to the PHC clinics and local hospitals. Subsequently, PC as a formal initiative has expanded into other subdistricts in the region, namely Oudtshoorn and Mossel Bay. Knysna and Bitou subdistricts already were offering local PC services. Increased awareness of PC has been facilitated through provincial PC training courses for all cadres of healthcare workers. District-wide groups of doctors, nurses, allied healthcare workers and community healthcare workers received face-to-face training in interprofessional workshops over 5 days on several occasions. Furthermore, online PC training courses, continuous medical education sessions and bedside teaching with students and health carers expanded awareness.

Recognising the growing development and funding focus on PC in South Africa, this article describes a rural PC model, which is integrated into the overall health service. We describe the current service, identify factors that contribute to sustainability and efficiency, highlight examples of good practice and identify areas for further development.

### Aim

The aim of this research was to review the PC project from November 2018 to November 2022. The objectives included describing the current PC service, quantifying the number of patient referrals, describing the number of home visits, quantifying the hospital PC ward rounds and making recommendations to strengthen the PC service.

## Research methods and design

### Study design

This was a retrospective descriptive study.

### Setting

The study was conducted in the George subdistrict of the Garden Route district in the Western Cape Province of South Africa. George has an estimated population of 218 381.^10^ While the subdistrict has a high level of socioeconomic development, there are large areas such as the Thembalethu suburb (population approximately 50 806) where the unemployment rate is estimated at 14.3%, with 17.3% of households situated in informal settlements.^11^ Community-based services are offered to all patients with PC needs by three NGOs. The NGOs employ community health workers (CHWs) and deliver home community-based care (HCBC) in the area. Twenty-eight CHWs are in the service of an NGO with a strong hospice and PC background. The local branch of a national NGO specialising in family support employs 12 CHWs, and an NGO initially established to care for HIV/AIDS and tuberculosis patients employ a further 30 CHWs. These organisations are supplemented by 3 community day centres, 10 fixed and 4 mobile PHC clinics, 1 intermediate care facility that also provides inpatient PC, as well as 1 regional and 1 tuberculosis hospital. The regional hospital is a 266-bed general specialist hospital, with a 23-bed family medicine ward, as well as the usual general specialties. The private sector offers limited formal PC to patients.

### Study population

All patients in the George subdistrict referred for PC to the government health services from November 2018 to November 2022 were included.

### Sampling and data collection

A database is kept in an Excel spreadsheet via a password-protected online software programme (Microsoft OneDrive)^©^ where all patients referred for PC are entered into. Previously, this was maintained by the subdistrict physiotherapist and subsequently continued by the PC doctor.

All PC referrals from the regional hospital, PHC clinics and local TB hospital are made via a standardised referral form to a secure group email address dedicated to PC. All patients entered into the database and all patients referred to the email group were sampled. Data were initially collected for clinical rather than research purposes, and therefore a pragmatic approach to data collection was used, with additional variables added as the service grew. Variables included patient demographics (age, sex and area of residence), the diagnosis, the validated Supportive and Palliative Care Indicators Tool (SPICT) classification (see Appendix A),^12,13^ the number and origin of referrals made, the number of home visits made, the reason for the home visit and by which healthcare professionals and the date and place of death. Data from PC hospital ward rounds were collected via the EpiCollect5© app. These data were retrospectively analysed.

### Data analysis

Quantitative data were analysed using descriptive statistics including means and standard deviations to describe continuous variables that were normally distributed. Categorical data were analysed using frequencies and percentages.

### Ethical considerations

Ethics approval was obtained from the Stellenbosch University Higher Research Ethics Committee (ethics reference number N22/09/116) and the Provincial Department of Health Research Ethics Committee (reference number WC_202301_013). Institutional approval was also obtained. All data were anonymised.

## Results

### The current palliative care model

Patients are referred from the regional hospital wards, outpatient’s department or emergency centre (EC), the tuberculosis hospital, the PHC clinics and occasionally from the private sector to the PC service via a one-page referral document, emailed to a secure PC group email address. The PC email group constitutes a small number of doctors, nurses, NGO coordinators, social workers and physiotherapists. As the PHC clinic drainage area of the referred patient is expected in the subject line of the email, different people in the email group are responsible for different areas and respond accordingly to the referral. The response includes a home visit, a ward round or a telephone call to the patient’s home. The sessional PC doctor maintains the patient database in an Excel spreadsheet in a secure online folder.

Weekly regional hospital multidisciplinary team (MDT) ward rounds take place on Wednesday afternoons, to review referred patients while they are inpatients. Home visits take place daily. The first visit is usually undertaken by a sessional PC doctor and/or a PHC nurse from the NGOs, with a plan for subsequent visits made based on the patient’s needs. Further visits are undertaken at varying frequencies and by different members of the MDT depending on the patient’s needs. Telephonic follow-up also occurs. Some residential areas have a stronger service presence than others.

Palliative care ward rounds at the intermediate care facility take place 3–4 times per week, led by the sessional PC doctor, sometimes with a medical student or a junior doctor. Further informal telephonic consultations and home support by CHWs and nurses occur in the community, often after-hours. Currently, this work is often not formally documented.

Finally, regular strategic planning and review meetings with PC stakeholders take place every 2 months at the regional hospital, governed by the Department of Family Medicine. These stakeholders include the heads of family medicine, physiotherapy, social work and oncology; clinicians; the PC nursing sister; NGO training coordinators; PHC nursing and PHC medical services.

Palliative care training has been prioritised. District-wide awareness around PC is actively maintained through regular training of staff, continuing medical education sessions and bedside and formal undergraduate and postgraduate student teaching. Accredited training has been provided through 5-day short courses in face-to-face and online formats, managed by the provincial people development centre. The sessional PC doctor has also trained the CHWs in PC. During the twice-daily handover ‘huddle’ in the EC at the regional hospital, all patients with PC needs in the unit or the shift doctors admitted to the family medicine ward are discussed and assigned an ICD-10 PC code (Z51.5).

### Referrals

A total of 819 patients have been referred to the PC team during the study period. Inpatient referrals were reviewed by the PC team during the weekly PC ward rounds. As patients were discharged, the referrals were sent via a group email address to the relevant NGO coordinator, who arranged home visits for patients, as necessary.

**FIGURE 1:**
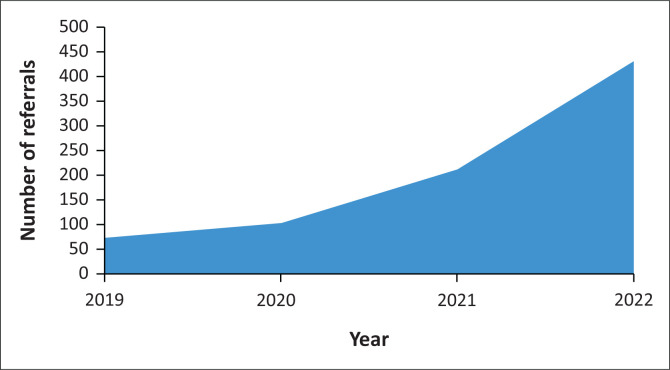
Number of referrals per year.

The number of patients has increased steadily over the study period. In 2019, 64 patients were referred. In 2020, 124 patients were referred. In 2021, 214 were referred and by 30 November 2022, and 431 had been referred for the year 2022. This represented an average referral increase of 89% per year (see Figure 1).

**FIGURE 2:**
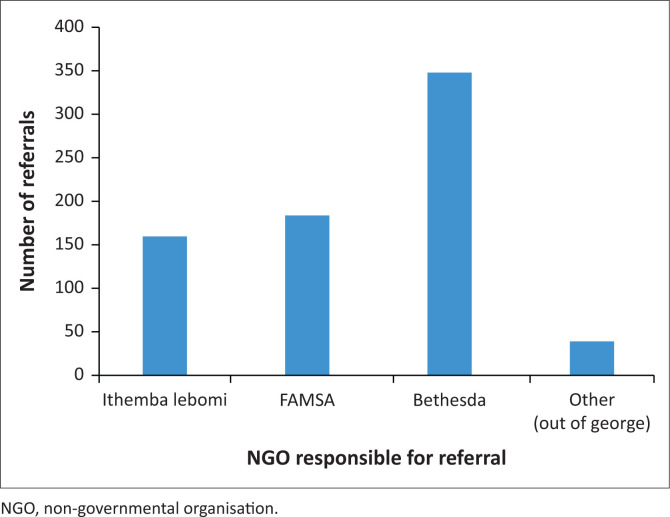
Referrals by non-governmental organisation.

Referrals were made for patients living in 17 residential areas. The greatest number of referrals were for patients living in the residential area of Thembalethu (161 referrals), followed by patients living in the Pacaltsdorp residential area (127 referrals). When grouped by NGO responsible for the home visit (see Figure 2), Bethesda, the NGO responsible for the Greater George, Uniondale and Herold, had the most referrals.

In total, 87% of referrals were made from the local regional hospital, and only 6% were made by the local PHC clinics or other PHC services. Other referral sources included 15 referrals directly from the community, via CHWs or the NGO nurses, 27 from other hospitals in the region including the TB hospital, 5 from the private sector and 2 from frail care institutions. The average age of patients that were referred was 61 years, with an age range from 1 to 83 years. Twelve children were referred to the PC team.

Referred patients were classified according to the SPICT criteria (see Appendix 1), modified and validated for use in South Africa.^12,13^ The most common diagnosis of referred patients was cancer (57%). Eight percent had a diagnosis of kidney disease, 8% neurological disease including cerebrovascular accidents, 8% lung disease, 7% infectious disease, 6% frailty or dementia, 6% heart or vascular disease and 1% liver disease. No patients were recorded as having been referred with a primary diagnosis of trauma or haematological disease.

### Home visits

While the number of home visits was not part of initial routine data collection, manual analysis of the data revealed that at least 152 home visits have been recorded since the programme began. This number is likely to be an underestimate as many home visits were carried out informally prior to the introduction of obligatory data capturing. Home visits have become part of routine data collection, and in November 2022, 38 home visits were carried out. Seventeen were attended by a nurse, 10 by a social worker, 8 by a doctor and 8 by a CHW.

Since the programme began, 46% of patients were recorded as having died at home; however, this percentage has been increasing as the scope of home-based PC has increased in the community. For example, in November 2022, 79% of patients died at home, in accordance with their choice. This was seen as a positive indicator of the programme as it reduced the burden on hospital care and it capacitated families and carers of patients.

**FIGURE 3:**
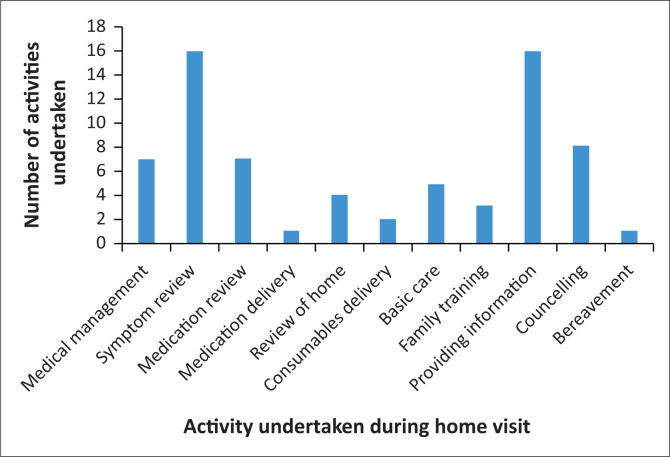
Activities during home visits in November 2022.

The activities carried out by healthcare professionals during home visits were wide-ranging (see Figure 3). Reviewing symptoms and providing information were the two most common activities; however, other services provided included the prescription, adjustment and delivery of palliative medication and consumables, reviews of homes, basic care provision and family training, as well as counselling, both for the patient and the family. The demands for consumables were increasing, as increasing numbers of patients with PC needs were referred.

### Regional hospital multidisciplinary team ward rounds

A total of 122 MDT hospital ward rounds took place during the study period. During these ward rounds, 345 patients were reviewed by the PC team, with an average time of 75 min spent per ward round. The ward rounds were well attended by members of the MDT. On average, 95% of rounds were attended by a PC specialty doctor, 91% by a social worker, 82% by a physiotherapist and 30% by a psychologist. The team emphasised MDT care and ownership of the patient by the ward team. A representative doctor from the patient’s ward team attended 79% of rounds. This ensured ongoing bedside teaching in PC, creating understanding and built capacity for PC. In addition, 30% of rounds were attended by the patient’s ward nurse. During the ward rounds, a patient-centred approach was used, which allowed for in-depth understanding of the patient’s symptoms, concerns and expectations. As far as possible, the ward rounds included the patients’ families, for shared decision-making. A PC plan was checked with the patient and family (see Appendix 2).

## Discussion

The current PC model has evolved from a pilot project^9^ in 2018 to an established, integrated service component of the subdistrict health system. A key factor for the success and sustainability of the service has been local champions of PC continuously advocating for improved PC services. The appointment of a sessional PC doctor by the regional hospital management in the Department of Family and Emergency Medicine provides agency, continuity and advocacy. Furthermore, a team of doctors in family and emergency medicine and in PHC, PHC nurses, CHWs, allied healthcare colleagues including physiotherapists, social workers and psychologists and managers created a network of PC providers that allowed the project to become embedded in the health services. Dedicated and committed staff are known to positively influence awareness and attitudes towards PC, leading to a ‘bottom-up’ drive for improvement and change, alongside ‘top-down’ direction from management and policy drivers.^14^ An example of this can be found in the Abundant Life Palliative Care programme at Victoria Hospital in Cape Town.^7^ A strong component of the programme has been to engage with community resources, working closely with the local NGOs alongside the public healthcare system.

While the programme was launched as a specific pilot project, care was taken from the beginning that it was not to be a standalone, vertical initiative, but an integrated component of the public health system. There was a focus on shared care of patients and reducing specialty silos, with the aim of every doctor feeling confident to manage patients with PC needs. It has been shown that integrating PC services leads to improved symptom control, reduced use of ineffectual disease-focused treatments at the end of life, improved family satisfaction, improved quality of life and efficient use of healthcare resources.^15^ Palliative care concepts are applicable to every specialty, and throughout the disease course, from diagnosis of a life-threatening illness through to end-of-life care and bereavement support.^16^ It was therefore imperative that all healthcare professionals should be proficient in the provision of PC as well as disease-specific treatment.^17^ Subdistrict-wide awareness around PC was maintained through regular training of all health staff in the hospitals and clinics, continuing medical education and regular bedside and formal undergraduate and postgraduate teaching. Within the regional hospital, there was a good understanding of PC principles, and the patients presented during the weekly PC ward rounds remained the responsibility of the referring hospital ward team.

Another success factor that contributed to the effectiveness and feasibility of the PC service was the use of simple tools. A significant barrier to PC provision is the identification of patients with PC needs. The SPICT tool was used to encourage the identification of suitable patients.^12^ The one-page PC plan also served as a referral letter, for follow-up in the community (see Appendix 2). A central provincial group email address served to facilitate efficient referral pathways and follow-up. The two data collection tools EpiCollect5^©^ and a secure, shared Microsoft OneDrive file ensured data availability that was regularly reviewed and acted upon to identify and address systems issues.

Most referrals were made by staff from the regional hospital, with only a small percentage coming from the PHC facilities. Anchoring the PC model initially within the regional hospital provided a solid starting point for the programme and was seen as a strength. However, the PC needs emanating from the many communities around the regional hospital were most likely underrepresented, as reflected in the low referral rate from communities and PHC services. Anecdotally, it seemed most presentations occurred at a late stage in the disease, ‘necessitating a hospital visit’ from where most referrals were then made. Previous work supports this late presentation of patients with PC needs, with factors including poverty and patient beliefs about illness playing a role.^17^ It is important to understand the factors influencing referral practices, as they impact on access and service utilisation.^18^ Knowledge of and trust in the PC service provided can increase referral rates, as does increased referrer training in PC.^16,18^ During the study period, the number of referrals grew by an average of 89% per year. One possible reason for this could be the PC training and an increasing awareness and acceptance of PC principles among hospital staff.

Home-based PC supported patients in their own homes, reducing the need to attend healthcare facilities. Home visits enabled patients to be reviewed medically, including the assessment of symptoms and prescription and delivery of medication and consumables, and offered both practical and psychological support for patients and their families. Family members shared decision-making with the healthcare workers who visited them, who provided advice and support to empower families as caregivers, which in turn ensured that the patient had access to quality care. Home-based PC can improve the likelihood of a patient dying in their preferred location.^19^ This can reduce healthcare costs and improve resource utilisation, reduce EC visits and shorten average lengths of hospital stay.^19,20^ A recent study in the regional hospital EC found that patients with PC needs made up 4.24% of EC attendances, with symptom control being the most common reason for attendance.^21^ This article highlighted the need for optimisation of home-based PC in order to alleviate hospital pressures and improve the experience of patients with life-limiting conditions.

The PC programme depended heavily on the community services, offered through the three NGOs in liaison with the PHC clinics. With about 70 CHWs for 218 000 population, this service is underresourced.^22^ There are also uneven service requirements and provision in different areas. For example, the Thembalethu residential area, with an estimated population of 50 806, was the single area with the greatest number of referrals. This area is served by one NGO. A second NGO received the greatest total number of referrals but works over a wider geographical area. Further possible reasons for the disparity of service provision in different areas included differences in local NGO leadership, the presence of sufficient CHWs to reach the local population and differences in rates of PC training among CHWs. Accessibility and safety concerns associated with visiting certain residential areas also remain issues for CHWs.^22^

Furthermore, there remains uncertainty over the optimum structure of local healthcare services in order to best meet the needs of home-based PC patients.^22^ While PC provision is listed in the NPFSPC job description for CHWs,^4^ they focus more on preventative and promotive aspects of health, with very little scope for ‘basic care’, including PC.^22^ While 152 home visits were formally recorded, the study highlighted that many ‘informal’, often after-hours home visits were also conducted by PC nurses and doctors. These informal systems were unclear, inequitable and risked overburdening already busy individuals. Community health workers can meaningfully contribute to the provision of many aspects of PC.^22,23^

The MDT hospital PC ward rounds have continued before, during coronavirus disease 2019 (COVID-19) (although much diminished) and after the COVID-19 epidemic. Of note was the ongoing multidisciplinary nature of the ward rounds. It is recognised that an MDT facilitates the coordination of clinical and social services and therefore best addresses the complex needs of patients and their families.^24^ As more doctors and nurses have undergone training in PC, there has been a refocus on how patients were referred. All healthcare professionals should aim for competence in providing PC alongside disease-specific treatment.^17^ Referrers were asked to consider the exact indication for referral and to what extent had an end-of-life conversation taken place by the attending ward team, with the patient and the family. This is a focus area that needs further attention to optimise the role of MDT ward rounds and encourage shared ownership of patients with palliative needs, as the need for PC continues to grow.

### Recommendations

1. As the number of patients with PC needs continues to grow, with subsequent demand for more home visits increases, there is a growing need to increase resources. These would include increased sessions for a doctor with PC experience, a dedicated PC nurse bridging the hospital, PHC clinics and community, more CHWs and more consumables.
2. The role of the CHWs in providing PC should be formalised and supported with training in PC. To optimise PC services, the NGOs need strengthening, as they look after most patients in the community. Some of the possible areas of strengthening include training the CHWs in PC, releasing a PC nurse to join them on home visits and equipping the CHWs with mobile technology and data to communicate easier with a PC nurse or doctor.
3. Improve data collection on an ongoing basis through simple tools. These must include the home visits and subsequent course of the patients’ illness experience. Capturing data about PC is essential and facilitates opportunities for research and advocacy to increase the scope of PC in the region.^7^ Data collection tools have been simplified, made more accessible and implemented to ensure that going forward, data are available to inform resource planning and service delivery, as well as to minimise patients lost to follow-up and to improve community and stakeholder support for expanding home-based PC.
4. Make a contact telephone number available for the families of patients with PC needs. No such service exists, forcing families and patients to phone the ambulance service and access the hospital or clinics. To optimise home-based PC, alleviate inappropriate attendances to the hospital EC and improve the experience of patients with life-limiting conditions, it is important that patients have access to a 24-h contact telephone number. This contact number will be used to address questions that patients and their caregivers may have and to enable signposting to relevant healthcare services.
5. Facilitate referrals earlier, from the community and from PHC clinics to PC services. This could happen through capacitating CHWs and clinics staff to recognise patients earlier and proactively make a shared management plan, including symptom control, with families and patients.
6. Strengthen the MDT approach to PC. Continue to include hospital, clinic and community staff, in order to create a seamless referral pathway. The role of the clinic pharmacists in opioid prescriptions, paediatric doctors in children with PC needs and utilising community members and other NGOs must be recognised and optimised.

### Study limitations

The retrospective data used were incomplete, with some records of patients not kept up-to-date throughout the study period. Typical data that were missing from some patient records included whether a home visit occurred or whether the patient passed away at home or in the hospital. The authors minimised these limitations by manually trolling through the data and correlated findings with the MDTs.

## Conclusion

The aim of this research was to review the PC service in a rural subdistrict in South Africa over the last 4 years. Despite many ongoing challenges, the programme has become sustainable and integrated in the public health system. The findings from this work may be useful to PC programmes in similar contexts elsewhere.
